# Insight into the antioxidant activities of ten Fabaceae plant species that are medicinally used by the Aucan Tribal Peoples from the Republic of Suriname (South America)

**DOI:** 10.30574/gscbps.2022.20.3.0349

**Published:** 2022-09

**Authors:** Dennis R.A. Mans, Nicholaas M. Pinas, Meryll Djotaroeno, Priscilla Friperson, Jennifer Pawirodihardjo, Maureen Y. Lichtveld

**Affiliations:** 1 Department of Pharmacology, Faculty of Medical Sciences, Anton de Kom University of Suriname, Paramaribo, Suriname.; 2 Department of Environmental Health Sciences, School of Public Health, University of Pittsburgh, Pittsburgh, USA.

**Keywords:** Fabaceae, Traditional medicine, Aucan Tribal Peoples, Suriname, Antioxidant activity, Total phenolic content

## Abstract

Fabaceae are associated with a high antioxidant activity (AA) and a high total phenolic (TPC), total flavonoid (TFC), and selenium content (SeC). In this study, the aqueous extracts from ten Fabaceae species that are medicinally used by the Aucan Tribal Peoples from Suriname (South America), were evaluated for AA using a DPPH and a FRAP assay, and for TPC, TFC, and SeC using Folin-Ciocalteu’s, an AlCl3 colorimetric, and an azure B-based method. Associations between pairs of these variables were determined by Pearson correlation coefficient. One-way ANOVA with post-hoc Tukey’s test was used to evaluate the data for statistically significant differences (p < 0.05). The *I. stipularis* (bark), *C. guyanensis* (bark), *A. jupunba* (twigs), and *M. urens* (fruit) extracts had the highest DPPH IC_50_ values (36 – 70 μg/mL) and FRAP values (346 – 573 μM FeE/100 μg) and the highest TPC (25 – 41 GAEq/100 μg), TFC (21 – 39 REq/100 μg), and SeC (4 −17 μg/g). The values for the *T. indica* (leaf), *P. macroloba* (bark), *M. pigra* (whole plant), *S. quinquangulata* (leaf), *A. sensitiva* (whole plant), and *L. leucocephala* (leaf) extracts were > 10-fold lower. AA, TPC, TFC, and SeC correlated well with each other (correlation coefficient ≥ 0.83, p ≤ 0.0030). Thus, AA, TPC, TFC, and SeC may represent important determinants of the health benefits of the former four samples but not of the others. Future studies should focus on the precise contribution of AA, TPC, TFC, and SeC to the therapeutic value of medicinal Fabaceae.

## Graphical Abstract

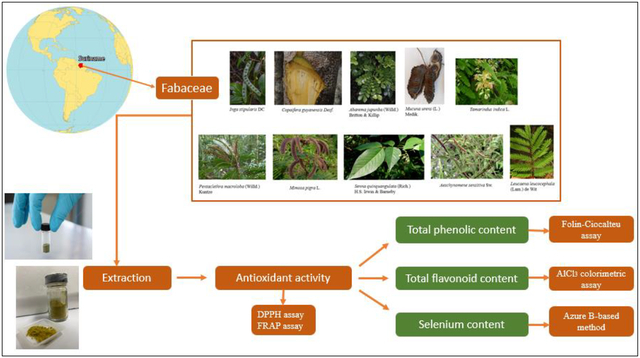

## 1. Introduction

Reactive oxygen species (ROS) are derivatives of molecular oxygen such as peroxide, superoxide, hydroxyl radical, and singlet oxygen [1]. ROS are continuously formed in metabolic reactions involving oxygen, such as the mitochondrial electron transport chain where ATP is produced [2] and the oxidative burst in activated phagocytic cells when ingested invaders and cell debris are destroyed [3]. ROS are also produced following inhalation of air pollutants such as ozone, nitrogen dioxide, particulates, car exhaust and cigarette smoke; alcohol intake; exposure to ionizing radiation; the consumption of diets high in fat, sugar, and processed products; the use of certain medical drugs such as the antineoplastic agents cyclophosphamide and doxorubicin; and during chronic infections and inflammatory disorders [4].

At non-toxic levels, ROS fulfill important functions in normal metabolic processes such as redox signaling, where they transduce signals for key cellular functions including differentiation, tissue regeneration, and longevity [5]. However, when in excess, ROS readily react with and inflict damage to DNA, RNA, lipids, proteins, and co-factors of specific enzymes [6]. The resulting oxidative stress leads to the disruption of cellular metabolism, eventually causing, among others, cardiovascular, diabetic, neoplastic, neurodegenerative, age-related, and inflammatory diseases [7].

Fortunately, the body possesses several defense mechanisms to cope with oxidative stress, including enzymatic antioxidant systems such as superoxide dismutase, catalase, and glutathione peroxidases [8] and non-enzymatic systems such as bilirubin and albumin [9]. In addition, exogenous antioxidants provided through the diet may help protect the body from oxidative stress and associated health problems [10]. Well-studied examples are phenolic compounds such as phenolic acids, flavonoids, anthocyanins, and tannins [11] along with vitamins such as ascorbic acid and α-tocopherol, β-carotene, polyunsaturated fatty acids, and essential minerals such as selenium [12]. These substances prevent the formation of ROS or interrupt their propagation, scavenge ROS, remove intermediates of ROS in redox reactions, inhibit ROS-generating oxidation reactions, and repair oxidized molecules [8–12].

When considering the critical role of dietary antioxidants in the mitigation of oxidative stress, the consumption of fruits and vegetables that are rich in these compounds has been recommended in order to decrease the risk of developing the above-mentioned degenerative diseases [13]. The pea family or Fabaceae (also known as Leguminosae) is a large family of flowering plants [14] that comprises many edible and highly nutritious species of major industrial importance such as the soybean *Glycine max* (L.) Merr. and the peanut *Arachis hypogaea* L. [15]. Species of Fabaceae are probably also among the most common ingredients of traditional medicinal preparations of indigenous and rural peoples throughout the world [16–19].

The widespread use of Fabaceae species as staple foods and traditional medicines has particularly been attributed to their high content of phenolic compounds with powerful antioxidant properties [20–23]. For example, this has been reported for nine Fabaceae species growing in Serbia and Montenegro [24], twenty-nine mature raw varieties of grain legume seeds produced in Europe [25], a number of under-utilized food legumes consumed in China [26], and the edible bean extracts of fifty Fabaceae populations grown in Thailand [27]. In addition, the Fabaceae presumably constitute the greatest number of species known to hyperaccumulate selenium, *i.e*., take selenium up from the soil and store it in their cells at concentrations of over 1,000 mg per kg dry weight [28]. Selenium is an essential micronutrient that serves, among others, as a building block of the amino acid selenocysteine that is part of various selenoproteins including glutathione peroxidases [29].

The Republic of Suriname is located in South America at the Atlantic Ocean and next to French Guiana, Brazil, and Guyana. The country’s land area of about 165,000 km^2^ can roughly be distinguished into northern plains and a large southern interior with mostly dense, pristine, tropical rain forest [30]. Suriname is renowned for its ethnic diversity that includes, among others, the descendants from enslaved Africans (called Tribal Peoples) who have formed several independent tribes in the hinterland of the country [31]. Like all ethnic groups of Suriname, the Tribal Peoples have largely preserved their beliefs, values, language, religion, and specific ethnopharmacological traditions since their arrival in Suriname in the 17^th^ century, and the use of traditional medicines is deep-seated in each of the tribes [32]

Species of Fabaceae are encountered throughout the entire territory of Suriname, from the coastal plains all the way to the southern forested interior [33]. Several of these plants are extensively used as medicinal herbs for treating a variety of illnesses in the country [34, 35]. Whether this extensive traditional use may also be related to the abundance in these plants of antioxidant compounds such as phenolic compounds has not been investigated before. In the current study, a number of species of Fabaceae that are commonly used as traditional medicines by the Aucans, a tribe of Tribal Peoples living in the north-eastern part of Suriname, has been evaluated for their *in vitro* antioxidant activity and total phenolic content. Since flavonoids represent an important class of antioxidant plant phenolics [36] and given the status of species of Fabaceae as selenium hyperaccumulators [28], the plant samples have also been evaluated for their total flavonoid and selenium content. The results obtained may provide indications as to whether the broad medicinal use of these species of Fabaceae by the Aucans may be attributed - at least in part - to a relatively high antioxidant activity of the plants and to the involvement of their total phenolic, total flavonoid, and selenium content in this activity.

## 2. Material and methods

### 2.1. Plant selection, plant collection, and preparation of plant extracts

The species of Fabaceae evaluated in the current study are given in Table 1. Parts of the plants were collected in March 2019, mainly in and around the city of Moengo, which is located in the east-southern part of the rural-coastal district of Marowijne (GPS coordinates of 5° 36’ 29” N and 54° 23’ 55” W). The collection sites had been free of herbicidal or pesticidal use for at least the preceding six months. The collected plant parts are widely used as traditional medicines by the local Aucan community and were selected on the basis of information from community members who are knowledgeable about their medicinal properties mentioned in Table 1.

The plants were collected in close collaboration with the National Herbarium of Suriname (BBS) that is in the possession of a collection permit from the Surinamese Ministry of Physical Planning, Land and Forestry Management. At the time of the collections, none of the plants were on the International Union for Conservation of Nature’s Red List of endangered or threatened species [37]. The collected samples were authenticated by a staff member of the BBS, and voucher specimens were prepared and stored at that institution for future reference (Table 1).

The collected plant parts were thoroughly washed with distilled water, dried in open air, washed again, and extracted with water at 60 °C or 100 °C (Table 1). This was based on information from local experts about the methods of processing of the samples, *i.e*., by extracting, brewing, or boiling them with water in order to obtain medicinal teas, infusions, or decoctions. The extracts were filtered, freeze-dried, divided in aliquots of 0.5 g, and stored at −20 °C until experiments.

### 2.2. Drugs and chemicals

1,1-Diphenyl-2-picrylhydrazyl (DPPH), iron(III) chloride hexahydrate (FeCl3.6H2O), iron(II) sulfate heptahydrate (FeSO4.7H2O), 2,4,6-Tris(2-pyridyl)-s-triazine (TPTZ), Folin-Ciocalteu reagent, gallic acid, aluminum chloride hexahydrate (AlCl3.6H2O), rutin, azure B, and sodium hydrogen selenite (NaHSeO3) were from Sigma-Aldrich (St. Louis, MO, USA). Ethanol was from Applichem GmbH (Darmstadt, Germany), sodium carbonate (Na2CO3) from Merck, (Darmstadt, Germany), and sodium acetate (CH3COONa) from BDH Laboratory Supplies (Poole, UK). All other chemicals used were from our laboratory stock and were of the highest grade available.

### 2.3. Determination of DPPH free radical-scavenging activity of plant extracts

The plant extracts were assessed for their antioxidant activity using a DPPH free radical scavenging assay [38]. This assay is based on the ability of an antioxidant to inactivate the stable DPPH cation free radical following donation of an electron or hydrogen atom. During this process, the violet-colored DPPH molecule becomes colorless to pale yellow, and this change can spectroscopically be monitored. Thus, for each plant extract, seven serial dilutions between 1 and 3,000 μg/mL were prepared, and 0.3 mL of each dilution was mixed with 3 mL absolute ethanol and 0.5 mL DPPH solution of 0.5 mM in ethanol. After 90 min in the dark and at room temperature, the absorbance of the solutions was measured at 517 nm against a mixture of 3.3 mL ethanol and 0.5 mL sample as a blank. The control solution consisted of 3.5 mL ethanol and 0.3 mL DPPH solution.

The percentage antioxidant activity (AA %) of each dilution of each plant extract was determined using the formula:

$$\text{AA \%}=100-\left|\frac{\left(Abs_{\text{sample}}-Abs_{\text{blank}}\right)\times 100}{Abs_{\text{control}}}\right|$$

Where:

Abs_sample_ is the absorbance of the plant extract

Abs_blank_ the absorbance of the blank

Abs_control_ the absorbance of the control.

For each plant extract, the absorbance values of the dilutions were plotted against the corresponding concentrations. From the resulting dose-response curve, IC_50_ values were derived, *i.e*., the concentrations of the plant extracts (in μg/mL) accomplishing a 50% decrease in absorbance when compared to untreated controls. The lower the IC_50_ value, the higher the antioxidant activity.

### 2.4. Determination of ferric reducing antioxidant power of plant extracts

The ferric reducing antioxidant power (FRAP) of the plant extracts was also determined by a spectrophotometric method, but this time based on the ability of an antioxidant to reduce a ferric (Fe^3+^) ion from the Fe^3+^-TPTZ complex to the ferrous (Fe^2+^) ion from a Fe^2+^-tripyridyltriazine complex through the donation of an electron at low pH [39]. The reactions were spectrophotometrically monitored by measuring the change from the colorless Fe^3+^-TPTZ complex to the intensely blue-colored Fe^2+^-tripyridyltriazine complex.

Thus, each freeze-dried plant extract was diluted to a concentration of 100 μg/mL, and 100 μL of these dilutions were mixed with 3 mL freshly prepared FRAP reagent and 1 mL distilled water. The FRAP reagent consisted of TPTZ 10 mM in HCl, FeCl_3_.6H_2_O 20 mM, and acetate buffer 300 mM pH 3.6 in the proportion of 1/1/10 (*v/v/v*). After thorough mixing and incubation for 30 min in the dark and at room temperature, the absorbance at a wavelength of 593 nm was recorded against a blank consisting of samples where the plant extract was substituted by distilled water. The change in absorbance was directly related to the total reducing power of the electron-donating antioxidants present in the plant extracts. These were estimated from a calibration curve constructed from the absorbance of different concentrations of FeSO_4_ at 593 nm and expressed as μM Fe^2+^ equivalents reduced per 100 μg lyophilized plant extract (μM FeE/100 μg).

### 2.5. Determination of total phenolic content of plant extracts

The total phenolic content of the plant extracts was determined using Folin-Ciocalteu’s method [40]. The Folin-Ciocalteu reagent is a mixture of phosphomolybdate and phosphotungstate, and the method is based on the transfer of electrons in alkaline medium from phenolic compounds to the phosphomolybdate/phosphotungstate complex to form a blue chromophore that is spectrophotometrically detectable. Thus, each freeze-dried plant extract was dissolved in distilled water to a concentration of 100 μg/mL. Of each extract, an aliquot of 1.0 mL was added to 0.1 mL Folin-Ciocalteu reagent 1 N, after which 0.9 mL distilled water was added. The mixture was shaken and allowed to react for 5 min at room temperature. Then, 1.0 mL of Na_2_CO_3_ 7% (*w/v*) was added. This solution was adjusted with distilled water to a final volume of 3.4 mL and thoroughly mixed. After incubation for 30 min in the dark, the absorbance was read at 765 nm with respect to a blank containing only Folin-Ciocalteu reagent 1 N and Na_2_CO_3_ 7% (*w/v*). The total phenolic content of the plant extracts was calculated from the linear equation of a standard curve prepared with gallic acid (1 to 200 μg/mL) and expressed as μM gallic acid equivalents per 100 μg lyophilized plant extract (μM GAE/100 μg).

### 2.6. Determination of total flavonoid content of plant extracts

The total flavonoid content of the plant extracts was determined using a previously described aluminum chloride colorimetric method [41]. This method is based on the formation of acid-stable complexes between AlCl_3_ and the hydroxyl groups of flavones and flavonols. Thus, each freeze-dried plant extract was dissolved in distilled water to give samples of 100 μg/mL. A volume of 0.5 mL AlCl_3_ 2% (*w/v*) in absolute ethanol was added to 0.5 mL aliquots of each sample, after which 0.5 mL 1 M potassium acetate and 0.5 mL 1 M HCL were added. The mixture was incubated for 10 min at room temperature and the absorbance was measured at 425 nm against a blank of distilled water. A yellow color indicated the presence of flavonoids. Total flavonoid content of the plant extracts was calculated by interpolation into a standard curve of rutin prepared from serial dilutions of this compound between 0 and 200 μg/L. Data were expressed as μM rutin equivalents per 100 μg lyophilized plant extract (μM RE/100 μg).

### 2.7. Determination of selenium content of plant extracts

The selenium content of the plant extracts was determined by a previously described spectrophotometric method using azure B as a chromogenic reagent [42]. This method relies on the reaction of selenium with potassium iodide in acidic medium to liberate iodine that bleaches the violet color of azure B, resulting in a decrease in its absorption that is linearly related to selenium concentration. Thus, 100 μg freeze-dried plant extract was digested with 1 mL concentrated HNO3 for 20 min and at a temperature of 140 ^o^C. After cooling, 1 mL of KI (2% *w/v*) and 1 mL of HCl 2 M were added, and the mixtures were gently shaken until they turned yellow, indicating the liberation of iodine. Next, 0.5 mL azure B (0.1% *w/v*) was added, the samples were shaken for 2 min, and diluted to give 10 mL by the addition of distilled water. Absorbances were measured at a wavelength of 644 nm and taken to correspond to the selenium content of the plant extracts after correction for values obtained with blanks prepared by replacing the plant extracts with distilled water. Selenium contents of the plant extracts were derived from a calibration graph curve constructed from serial dilutions of NaHSeO_3_ prepared between 0 and 200 μg/L. Data were expressed as μg selenium per g lyophilized plant extract (μg Se/g).

### 2.8. Statistics

All experiments were carried out at least three times in triplicate and data were expressed as means ± SDs. Possible correlations between DPPH free radical scavenging activity and ferric reducing antioxidant power, and between DPPH free radical scavenging activity or ferric reducing antioxidant power on the one hand, and total phenolic content, total flavonoid content, or selenium content on the other hand were explored by calculating the Pearson’s correlation coefficient. This value determines the strength of the linear relationship between two variables and has a value between −1 to 1, with a value of −1 meaning a total negative linear correlation, 0 indicating no correlation, and + 1 meaning a total positive correlation. In all cases, p values < 0.05 were taken to indicate statistically significant differences.

## 3. Results

### 3.1. Antioxidant activity

The aqueous extracts from ten species of Fabaceae that are medicinally used by the Aucan Tribal Peoples in the Surinamese rural-coastal districts of Marowijne (Table 1), were assessed for their antioxidant activity using a DPPH assay [38] and a FRAP assay [39]. The extracts from *I. stipularis* (bark), *C. guyanensis* (bark), *A. jupunba* (twigs), and *M. urens* (fruit) had the highest DPPH free radical-scavenging activities with IC_50_ values of 35.8 ± 1.4 to 70.0 ± 3.5 μg/mL (Table 2). These values did not differ statistically significantly from each other (Table 2). These samples also had the highest FRAP values (345.8 ± 6.6 to 573.2 ± 7.0 μM FeE/100 μg; Table 2) which differed statistically significantly but not much from each other (p < 0.0001; Table 2).

The antioxidant activities of the extracts from *T. indica* (leaf), *P. macroloba* (bark), *M. pigra* (whole plant), *S. quinquangulata* (leaf), *A. sensitiva* (whole plant), and *L. leucocephala* (leaf) were statistically significantly and substantially lower when compared to those from the above-mentioned four samples (p < 0.0001), occurring at DPPH IC_50_ values of 312.0 ± 34.0 to 653.0 ± 35.0 μg/mL and FRAP values of 7.3 ± 0.6 to 95.8 ± 7.1 μM FeE/100 μg (Table 2). There were also statistically significant differences among most of these values (p < 0.0001; Table 2). Applying Pearson correlation method, a strong, statistically significant negative association was found between the DPPH free radical-scavenging activity and the antioxidant power of the sample series (correlation coefficient of −0.880; p = 0.0008; Table 3).

### 3.2. Total phenolic content and total flavonoid content

Next, the plant extracts were assessed for their total phenolic content and total flavonoid content using Folin-Ciocalteu’s method [40] and an aluminum chloride colorimetric method [41], respectively. The results from these experiments are given in Table 4. The extracts from *I. stipularis*, *C. guyanensis*, and *A. jupunba* had the highest total phenolic content and total flavonoid content (37 ± 8 to 41 ± 8 μM GAEq/100 μg and 30 ± 5 to 39 ± 5 μM REq/100 μg, respectively). These values did not differ statistically significantly from each other and were roughly twice higher than those found for the *M. urens* extract (p ≤ 0.03975 and p ≤ 0.00753, respectively). Total phenolic and total flavonoid contents of the *T. indica*, *P. macroloba*, *M. pigra*, *S. quinquangulata*, *A. sensitiva*, and *L. leucocephala* samples were again much lower (between 3 ± 1 and 7 ± 1 μM GAEq/100 μg and between 2 ± 0 and 6 ± 4 μM REq/100 μg, respectively), also differing statistically significantly from the values found for the *I. stipularis*, *C. guyanensis*, *A. jupunba*, and *M. urens* samples (p < 0.0001).

Pearson correlation showed a statistically significant positive correlation between total phenolic content and total flavonoid content of the series (correlation coefficient of 0.99, p < 0.0001; Table 3). There was also a statistically significant positive correlation between DPPH IC_50_ values and total phenolic content (correlation coefficient of 0.88, p < 0.0001), and between FRAP values and total phenolic contents (correlation coefficient 0.95, p < 0.0001; Table 3). The same held true for the associations between DPPH IC_50_ and FRAP values of the series on the one hand, with total flavonoid content on the other hand (correlation coefficient of 0.88, p = 0.0009, and correlation coefficient of 0.94, p value < 0.0001, respectively; Table 3). Thus, the DPPH free radical-scavenging activity and the antioxidant power of the sample series was probably for an important part determined by the total phenolic and total flavonoid contents of the samples.

### 3.3. Selenium content

The plant extracts were also assessed for their selenium content using an azure B-based method [42]. As found for the DPPH free radical-scavenging activities and the FRAP values as well as total phenolic and total flavonoid contents, the extracts from *I. stipularis*, *C. guyanensis*, and *A. jupunba* had the highest selenium contents, namely 16.7 ± 1.8, 13.7 ± 2.2, and 12.2 ± 0.7 μg/g (Table 5). The selenium content of these samples differed statistically significantly but only slightly from each other (p ≤ 0.0632). The selenium content of the *M. urens* extract was about 4 times lower than that of the *I. stipularis* extract (Table 5; p < 0.0001). And that of the *T. indica*, *P. macroloba*, *M. pigra*, *S. quinquangulata*, *A. sensitiva*, *L. leucocephala* extracts was roughly 6 to 60 times lower than that of the *I. stipularis* extract (Table 5; p < 0.0001).

Pearson correlation indicated a good correlation between DPPH IC_50_ values and FRAP values on the one hand, and selenium content of the samples series on the other hand, with correlation coefficients of −0.83 (p = 0.0030) and 0.91 (p = 0.0003), respectively (Table 3). There were also good correlations between total phenolic content and selenium content (Pearson correlation coefficient of 0.95; p < 0.0001) and between total flavonoid content and selenium content (Pearson correlation coefficient of 0.96; p < 0.0001; Table 3). Apparently, the selenium content of the sample series also seemed to represent an important determining factor in the antioxidant activity and the antioxidant content of the sample series.

## 4. Discussion

The Fabaceae has the reputation of a plant family with a relatively high antioxidant activity and a relatively high total phenolic, total flavonoid, and selenium content [20–23]. In this study, we sought indications as to whether the broad traditional medicinal use of ten species of Fabaceae by the Surinamese Aucans may be associated with these properties. To this end, aqueous extracts from parts of the plants were evaluated for their antioxidant activity using a DPPH and a FRAP assay, after which the data obtained have been related to the total phenolic, total flavonoid, and selenium content of the samples. The preparations from *I. stipularis* (bark), *C. guyanensis* (bark), *A. jupunba* (twigs), and *M. urens* (fruit) had the greatest DPPH free radical-scavenging activity and FRAP values. These variables correlated well with the total phenolic, total flavonoid, and selenium content of the samples. The antioxidant activities as well as the total phenolic, total flavonoid, and selenium content of the extracts from *T. indica* (leaf), *P. macroloba* (bark), *M. pigra* (whole plant), *S. quinquangulata* (leaf), *A. sensitiva* (whole plant), and *L. leucocephala* (leaf) were substantially lower. Thus, the medicinal use by the Aucans of the former four species - unlike that of the latter six - may be supported, at least in part, by their relatively high antioxidant activity and relatively high contents of antioxidant constituents.

The relatively high antioxidant activity and total phenolic and flavonoid contents found for the samples of *I. stipularis* bark, *C. guyanensis* bark, *A. jupunba* twigs, and *M. urens* fruit were partially supported by literature data. *I. stipularis* leaf and several parts from other *Inga* species reportedly elicited strong antioxidant activity [43–45]. The antioxidant activity of *I. stipularis* leaf might be associated with its high content of flavonoids including the flavanonol astilbin [46], and that of the other *Inga* species with the presence of flavonoids, anthocyanins, tannins, and phenolic acids in the plants [43–45]. These compounds exerted antibacterial activity *in vitro* [47] and may be responsible for the presumed efficacy of several *Inga* species against injuries, pain, and inflammations by Amazonian indigenous communities [43, 46].

Literature data supporting the current observations with *C. guyanensis* bark, *A. jupunba* twigs, and *M. urens* fruit are scant. Several parts of *Copaifera* species reportedly contained appreciable amounts of phenolic compounds [48–50]. However, the relatively high antioxidant activity of these samples [50] has been attributed to terpenoids rather than phenolic compounds in the oleoresin of the bark [50], the main pharmacologically active substance of these plants [51, 52]. On the other hand, bark and leaf preparations of *A. cochliacarpos* (Gomes) Barneby & J.W.Grimes that is closely related to *A. jupunba*, exerted substantial antioxidant activities and had a relatively high content of phenolics, tannins, flavonols, and catechins [53, 54]. These compounds have been held responsible for the alleged efficacy of *A. cochliacarpos* against bacterial infections along with inflammatory disorders and wounds [55, 56]. And preparations from various species related to *M. urens* such as *M. gigantean* (Willd.) DC., *M. interrupta* Gagnep., *M. monosperma* Wight, and *M. pruriens* (L.) DC. displayed notable antioxidant activity and contained appreciable amounts of phenolic compounds [57, 58]. In the case of *M. pruriens*, these properties have been associated with antiparkinson and neuroprotective effects in animal models [59].

To our knowledge, there are no literature data to relate the current findings on the selenium content of the above-mentioned samples to. However, the good correlation between antioxidant activities and selenium content of the series suggest that selenium might play an important role in their antioxidant activity. This correlation can be attributed to the ability of selenium, similarly to phenolic compounds, to transfer a single electron in alkaline medium to molybdenum, contributing to the formation of the blue complexes formed by each of the plant extracts in the Folin-Ciocalteu assay [60, 61]. This was supported by the good correlation between total phenolic and selenium contents of the sample series. Apparently, the antioxidant activities of the samples were not only determined by their phenolic contents but also by their selenium contents. Comparable associations have been reported before for a number of medicinal plants from Lithuania, Poland, and Ukraine, for which the antioxidant properties were governed by both polyphenols and selenium [62].

Antioxidant activities and total phenolic, total flavonoid, and selenium contents of the other six plants also correlated well with each other, but these values were relatively low. However, these low values were not in accordance with literature data. Previous studies reported appreciable antioxidant activity and the notable presence of phenolic compounds (including flavonoids and their glycoside derivatives, phenolic acids, and tannins) in *T. indica* leaf [63, 64], the seed oil of *P. macroloba* [65] and the stem bark of the related oil bean tree *P. macrophylla* Benth. [66], parts of *M. pigra* and the closely related *M. pudica* [67–69], *S. quinquangulata* leaf and parts of several other *Senna* species [70, 71], parts of *A. sensitiva* and several other species in the genus *Aeschynomene* [72, 73], and *L. leucocephala* leaf [74, 75]. With the exception of *T. indica* leaf, there are also no literature data on the selenium content of this series of samples. However, the value of 2.6 ± 1.3 μg/g found for the *T. indica* preparation in the current study is in reasonable agreement with that of 4.7 μg/g that has previously been reported [64], giving credence to the current findings on the selenium content of this and the other plant samples.

The phenolic antioxidants in the plants addressed in the preceding paragraph have been associated with important pharmacological activities. For instance, a *T. indica* leaf extract, a *P. macrophylla* stem bark extract, a *M. pigra* whole-plant extract, and *Senna* preparations elicited antimicrobial activities [76–79], the aerial parts and the seed of *A. sensitiva* exhibited anti-inflammatory and antitumor activities *in vitro* [73], and *L. leucocephala* leaf exerted antidiabetic activity *in vitro* [75]. Notwithstanding, the reasons for the differences of the results from the current study with those mentioned in the literature are not clear. Possible explanations may include variations in species, strains, or varieties used; growing conditions of the plants; stages of maturity at which the plant parts have been harvested; the ways the plants have been processed; and/or the analytical methods applied to assess them for the various pharmacological activities [80–83]. These differences may well lead to dissimilarities in the concentration of certain phytochemicals including those with antioxidant activity [80–83], but this supposition must be verified in future studies.

Summarizing, the results from the current study are in line with literature data on the high antioxidant activity and total phenolic, total flavonoid, and selenium content of Fabaceae species [20–23] and support the traditional medical uses of *I. stipularis* bark, *C. guyanensis* bark, *A. jupunba* twigs, and *M. urens* fruit preparations by the Surinamese Aucans when considering the potential health benefits associated with these properties of the plants. Notably, the Aucan uses of these preparations against bacterial infections and asthma (Table 1) is in accordance with literature data on their pharmacological activities [47, 51, 52, 55, 56, 58]. The extracts of *T. indica* leaf, *P. macroloba* bark, *M. pigra* whole plant, *S. quinquangulata* leaf, *A. sensitiva* whole plant, and *L. leucocephala* leaf displayed relatively low antioxidant activities and total phenolic, total flavonoid, and selenium contents. This suggests that phenolic compounds, flavonoids, and/or selenium may not directly account for the medicinal use of these plants by the Aucans and that other pharmacologically active phytochemicals such as vitamins, steroids, alkaloids, saponins, and terpenoids may be involved in their beneficial effects including their antioxidant activity [72, 75, 84–87]. These considerations indicate the complexity of the precise relationships between antioxidant activity, total phenolic, total flavonoid, and selenium content of the plants on the one hand, and their health benefits on the other hand. Future studies should comprehensively address these relationships in order to obtain more scientific evidence on the specific contribution of these phytochemicals to the medicinal properties of the plants.

## 5. Conclusion

The results from this study suggest that the health benefits of four of ten species of Fabaceae that are commonly used by the Surinamese Aucans may be attributed, at least in part, to their relatively high antioxidant activity. This activity was probably for an important part determined by the total phenolic, total flavonoid, and selenium content of the plants. The relatively low antioxidant activity of the other six plant samples suggests that their presumed or apparent health benefits are determined by phytochemicals other than phenolic compounds. This argues against the characterization of the Fabaceae as “a plant family of antioxidant phenolic compounds”. These findings draw attention to the need to focus more on the precise contribution of antioxidant activity, total phenolic, total flavonoid content, and selenium content to the potential therapeutic value of medicinal Fabaceae and other medicinal plants in order to deploy implementation science strategies to assess and support the use of traditional herbal preparations.

## Figures and Tables

**Table 1:** Relevant information about the plants investigated in the current study. All reference vouchers have been stored at the National Herbarium of Suriname (BBS) at the Anton de Kom University of Suriname, Paramaribo, Suriname (UvS: University of Suriname)

Plant species (vernacular name in English; Surinamese)	Herbarium voucher	Part used; method of extraction	Traditional medicinal indication(s) according to local informants
*Inga stipularis* DC (sweet bean; switibonki)	UVS-18894	Bark; 30 min, 100 °C	Bacterial infections
*Copaifera guyanensis Desf*. (hoepel tree; opro udu)	UVS-18889	Bark; 30 min, 100 °C	Bacterial infections
*Abarema jupunba* (Willd.) Britton & Killip (soapwood; ingi sopo)	UVS-18898	Twig; 30 min, 100 °C	Bacterial infections
*Mucuna urens* (L.) Medik. (horse-eye bean; fayaston)	UVS-18895	Fruit; 1 h, 60 °C	Asthma
*Tamarindus indica* L. (tamarind; tamaren)	UVS-18899	Leaf; 1 h, 60 °C	Constipation, stomach ache
*Pentaclethra macroloba* (Willd.) Kuntze (oil bean tree; krubara)	UVS-18886	Bark; 30 min, 100 °C	::
*Mimosa pigra* L. (sensitive plant; sinsin tapu yu koto)	UVS-18892	Whole plant; 1 h, 60 °C	Constipation
*Senna quinquangulata* (Rich.) H.S. Irwin & Barneby (five-angular senna; yorkapesi)	UVS-18885	Leaf; 1 h, 60 °C	Stomach ache
*Aeschynomene sensitiva* Sw. (sensitive jointvetch; watrasinsin)	UVS-18896	Whole plant; 1 h, 60 °C	Hypertension
*Leucaena leucocephala* (Lam.) de Wit (river tamarind; pikin neku)	UVS-18902	Leaf; 1 h, 60 °C	Bacterial and fungal infections

**Table 2:** Antioxidant activities of the plant extracts investigated in the current study as determined by a 2,2-diphenyl-1-picrylhydrazyl (DPPH) free radical-scavenging assay and a ferric reducing antioxidant power (FRAP) assay

Plant species	DPPH IC_50_ values (μg/mL)	FRAP values (μM Fe^2+^ equivalents reduced per 100 μg lyophilized plant extract)
*I. stipularis*	37.0 ± 2.7^a^	492.0 ± 21.0^a^
*C. guyanensis*	35.8 ± 1.4^a^	573.2 ± 7.0^b^
*A. jupunba*	58.0 ± 3.4^a^	345.8 ± 6.6^c^
*M. urens*	70.0 ± 3.5^a^	363.3 ± 5.9^c^
*T. indica*	360.0 ± 8.7^b^	31.9 ± 10.4^d^
*P. macroloba*	410.0 ± 9.0^c^	41.2 ± 7.8^d^
*M. pigra*	540.0 ± 7.0^d^	95.8 ± 7.1^e^
*S. quinquangulata*	312.0 ± 34.0^e^	31.0 ± 9.0^d^
*A. sensitiva*	590.0 +7.5^f^	18.4 ± 3.1^f^
*L. leucocephala*	653.0 ± 35.0^g^	7.3 ± 0.6^g^

Values followed by different letters in superscript are statistically significantly different from each other (DPPH IC_50_ values: p ≤ 0.0022; FRAP values: p ≤ 0.0288)

**Table 3:** Associations among the various pharmacological activities and properties of the plant extracts investigated in the current study using Pearson correlation coefficient

	Pearson correlation coefficient	p value
DPPH IC_50_ values vs. FRAP values	−0.89	0.0008
Total phenolic content vs. total flavonoid content	0.99	< 0.0001
DPPH IC_50_ values vs. total phenolic content	0.88	0.0008
FRAP values vs. total phenolic content	0.95	< 0.0001
DPPH IC_50_ values vs. total flavonoid content	0.88	0.0009
FRAP values vs. total flavonoid content	0.94	< 0.0001
Total phenolic content vs. selenium content	0.95	< 0.0001
Total flavonoid content vs. selenium content	0.96	< 0.0001
DPPH IC_50_ values vs. selenium content	−0.83	0.0030
FRAP values vs. selenium content	0.91	0.0003

**Table 4:** Total phenolic and flavonoid contents of the plant extracts investigated in the current study as determined by Folin-Ciocalteu’s method and an aluminum chloride colorimetric assay, respectively

Plant species	Total phenolic content (μM GAEq/100 μg lyophilized plant extract)	Total flavonoid content (μM REq/100 μg lyophilized plant extract)
*I. stipularis*	41 ± 8^a^	39 ± 5^a^
*C. guyanensis*	37 ± 8^a^	30 ± 5^b^
*A. jupunba*	41 ± 7^a^	36 ± 5^a,b^
*M. urens*	25 ± 6^b^	21 ± 4^c^
*T. indica*	6 ± 1^c^	5 ± 1^d^
*P. macroloba*	5 ± 1^c^	3 ± 1^d^
*M. pigra*	7 ± 1^c^	6 ± 4^d^
*S. quinquangulata*	3 ± 1^c^	2 ± 0^d^
*A. sensitiva*	4 ± 1^c^	3 ± 1^d^
*L. leucocephala*	4 ± 1^c^	2 ± 1^d^

Values followed by different letters in superscript are statistically significantly different from each other (total phenolic content: p ≤ 0.0029; total flavonoid content: p ≤ 0.0075)

**Table 5:** Selenium content of the plant extracts investigated in the current study as determined by an azure B-based method

Plant species	Selenium content (μg/g lyophilized plant extract)
*I. stipularis*	16.7 ± 1.8^a^
*C. guyanensis*	13.7 ± 2.2^b^
*A. jupunba*	12.2 ± 0.7^b^
*M. urens*	4.1 ± 0.4^c^
*T. indica*	2.6 ± 1.3^c,d^
*P. macroloba*	2.1 ± 0.3^c,d^
*M. pigra*	1.2 ± 0.3^c,d^
*S. quinquangulata*	0.9 ± 0.3^d^
*A. sensitiva*	0.6 ± 0.1^d^
*L. leucocephala*	0.3 ± 0.1^d^

Values followed by different letters in superscript are statistically significantly different from each other (p ≤ 0.0309).
